# Effect of General Anesthesia Maintenance with Propofol or Sevoflurane on Fractional Exhaled Nitric Oxide and Eosinophil Blood Count: A Prospective, Single Blind, Randomized, Clinical Study on Patients Undergoing Thyroidectomy

**DOI:** 10.3390/jpm12091455

**Published:** 2022-09-05

**Authors:** Artemis Vekrakou, Panagiota Papacharalampous, Helena Logotheti, Serena Valsami, Eriphyli Argyra, Ioannis Vassileiou, Kassiani Theodoraki

**Affiliations:** 1 Department of Anaesthesiology, Aretaieion University Hospital, National and Kapodistrian University of Athens, 11528 Athens, Greece; 2 Department of Anaesthesiology, Achillopouleion General Hospital of Volos, 38222 Magnesia, Greece; 3 Hematology Laboratory—Blood Bank, Aretaieion University Hospital, National and Kapodistrian University of Athens, 11528 Athens, Greece; 4 Second Department of Surgery, Aretaieion University Hospital, National and Kapodistrian University of Athens, 11528 Athens, Greece

**Keywords:** fractional exhaled NO, sevoflurane, propofol, eosinophil blood count, thyroidectomy

## Abstract

**Background:** Nitric oxide (NO) is considered a means of detecting airway hyperresponsiveness, since even non-asthmatic patients experiencing bronchospasm intraoperatively or postoperatively display higher levels of exhaled NO. It can also be used as a non-invasive biomarker of lung inflammation and injury. This prospective, single-blind, randomized study aimed to evaluate the impact of two different anesthesia maintenance techniques on fractional exhaled nitric oxide (FeΝO) in patients without respiratory disease undergoing total thyroidectomy under general anesthesia. **Methods:** Sixty patients without respiratory disease, atopy or known allergies undergoing total thyroidectomy were randomly allocated to receive either inhalational anesthesia maintenance with sevoflurane at a concentration that maintained Bispectral Index (BIS) values between 40 and 50 intraoperatively or intravenous anesthesia maintenance with propofol 1% targeting the same BIS values. FeΝO was measured immediately preoperatively (baseline), postoperatively in the Postanesthesia Care Unit and at 24 h post-extubation with a portable device. Other variables measured were eosinophil blood count preoperatively and postoperatively and respiratory parameters intraoperatively. **Results:** Patients in both groups presented lower than baseline values of FeΝO measurements postoperatively, which returned to baseline measurements at 24 h post-extubation. In the peripheral blood, a decrease in the percentage of eosinophils was demonstrated, which was significant only in the propofol group. Respiratory lung mechanics were better maintained in the propofol group as compared to the sevoflurane group. None of the patients suffered intraoperative bronchospasm. **Conclusions:** Both propofol and sevoflurane lead to the temporary inhibition of NO exhalation. They also seem to attenuate systemic hypersensitivity response by reducing the eosinophil count in the peripheral blood, with propofol displaying a more pronounced effect and ensuring a more favorable mechanical ventilation profile as compared to sevoflurane. The attenuation of NO exhalation by both agents may be one of the underlying mechanisms in the reduction in airway hyperreactivity. The clinical significance of this fluctuation remains to be studied in patients with respiratory disease.

## 1. Introduction

Nitric oxide (NO) is a free radical in gas state, which plays an important role in a variety of processes relevant to respiratory physiology. The generation of NO follows both enzymatic and non-enzymatic pathways [1]. NO enzymatic production is catalyzed by three distinct isoforms of NO synthase: (i) neuronal NOS-1 (nNOS), mainly expressed in central and peripheral neurons, (ii) inducible NOS-2 (iNOS), expressed by many cell types as response to cytokines and other agents, and (iii) endothelial NOS-3 (eNOS), mostly expressed by endothelial cells. Non-enzymatic pathways, which are not clearly understood, produce NO through the reduction of NO₃ (nitrate) to NO_2_ (nitrite) [2]. An imbalance between iNOS and its constitutive isoforms (nNOS and eNOS) has been implicated in the pathophysiology of many cardiopulmonary diseases, since it can lead to excessive NO synthesis [3]. While physiological levels of NO possess anti-inflammatory properties, when increased due to the aforementioned upregulation of iNOS, NO becomes a pro-inflammatory mediator [2,4]. In fact, high concentrations of NO can be transformed into peroxynitrite radicals in the presence of oxygen-derived free radicals and play a significant role in the cellular damage associated with overproduction of NO [5].

Airways of patients with bronchial hyperreactivity may respond in an exaggerated way to a variety of stimuli, while airway instrumentation in such patients may lead to life-threatening bronchospasm, adding to the burden of morbidity this population may suffer in case they undergo general anesthesia for a surgical or diagnostic procedure [6]. Fractional exhaled NO (FeNO) has been used in the diagnosis of asthma, especially of the eosinophilic phenotype, and has also proved useful in guiding treatment of asthmatic individuals [7,8]. Additionally, non-asthmatic patients experiencing bronchospasm intraoperatively or postoperatively display higher levels of exhaled NO, a fact suggesting that the upregulation of the production of NO may play a role in airway hyperreactivity [9]. Increased levels of FeNO have also been found to correlate with sputum eosinophilia and eosinophilia in bronchoalveolar lavage fluid [10,11]. Furthermore, an increase in exhaled NO concentration has been used as an early marker of lung inflammation and injury in models of sepsis or acute lung injury induced by toxins [12,13]. Therefore, exhaled NO can be considered an efficient method for the prediction of airway hyperresponsiveness perioperatively, even in patients without known respiratory disease [14]. Additionally, it may be considered as an invaluable non-invasive biomarker reflecting early airway injury and inflammation.

Propofol, an intravenous anesthetic agent, can modify NO production by inhibiting the inducible production of NO in lipopolysaccharide-stimulated macrophages [15,16]. It has also been shown to exert protective effects in acute lung injury in experimental models [17,18]. There is also evidence that some intravenous anesthetics can influence chemotaxis of eosinophils in vitro [19]. Similarly, volatile anesthetics have been shown to attenuate the expression of inflammatory mediators and to alleviate bronchial hyperresponsiveness [20]. Sevoflurane-borne protection could also be mediated via the suppression of the iNOS/NO pathway, as decreased levels of NO metabolites have been demonstrated in plasma or lung perfusate of sevoflurane-pretreated rat models [20,21].

The variation of exhaled NO and eosinophils in surgical patients undergoing anesthesia has not been studied before. We hypothesized that there is a different effect of intravenous and inhalational techniques on the potential for airway hyperresponsiveness perioperatively, as this can be assessed by the measurement of exhaled NO and eosinophil blood count. If this is the case, it could also affect the selection of anesthetic maintenance techniques for patients with known hyperreactive airways. Therefore, the aim of the present study was to investigate the differential impact of two general anesthesia maintenance techniques on the exhaled NO and eosinophil blood count of patients without respiratory disease or airway hyperreactivity.

## 2. Materials and Μethods

### 2.1. Study Population

This is a prospective, single-blind, randomized, pragmatic trial, conducted between May 2014 and April 2018 in Aretaieion University Hospital, Athens, Greece. The protocol of the study was registered on www.clinicaltrials.gov (NCT02065635) (accessed on 30 April 2022). The study took place in compliance with the Helsinki Declaration, and its design was in accordance with the Consolidated Standards of Reporting Clinical Trials [22]. The study protocol was approved by the Ethics Committee of Aretaieion University Hospital, Aretaieion Hospital, National and Kapodistrian University of Athens, Chairperson Professor Ioannis Vasileiou on 19 December 2013 (B-19/19-12-2013).

After evaluating all eligible patients that were scheduled for thyroidectomy during this period, 60 patients were enrolled. Inclusion criteria were: age 18–75 years, all sexes, American Society of Anesthesiologists (ASA) physical status I-III, total thyroidectomy. Indications for thyroidectomy were: thyroid nodules, hyperthyroidism, substernal goiter, differentiated (papillary or follicular) thyroid cancer and medullary thyroid cancer. None of the patients presented with either clinical or radiological evidence for tracheomalacia. All patients were operated on by the same experienced surgeon and managed by the same anesthesiologist to avoid confounding. Exclusion criteria were: refusal or inability to consent due to language barriers or cognitive dysfunction, smoking, history of atopy, allergy, airway hyperresponsiveness or other respiratory disease, such as asthma and chronic obstructive pulmonary disease, contraindication to the administration of paracetamol, parecoxib or tramadol and treatment with nitrate medication. Informed consent for participation in the study was obtained at the preanesthetic visit.

Patients were randomized by a computer-generated list of random numbers (www.randomizer.org (accessed on 30 April 2022)) into two groups of different maintenance techniques: either inhalational anesthesia with sevoflurane (sevoflurane group) or continuous intravenous infusion of propofol 1% (propofol group).

### 2.2. Study Design and Anesthetic Management

On the operation day, patients undertook a fractional exhaled NO measurement using a portable analyzer of Nitric Oxide (NObreath^®^, Bedfont^®^ Scientific Ltd., Maidstone, UK) during the preanesthetic visit (T_0_). Patients had avoided the use of coffee during the last 12 h [23]. The NO measurement had to be performed at expiratory rates of 50 mL/s for 12 s and was expressed at parts per billion (ppb). If the patient exhaled outside the exhalation guidelines, the test failed. We complied with the American Thoracic Society suggestion for performance of two sequential measurements and calculation of their mean value for each patient [24]. Measurements with this portable analyzer have proven to be reliable in comparison to measurements performed with stationary analyzers [25].

On arrival to the operating room, standard monitoring (consisting of five-lead electrocardiography, pulse oximetry and non-invasive blood pressure measurement), peripheral nerve stimulator and Bispectral Index monitor (BIS) were applied. Additionally, intravenous access was secured and the first blood sample for eosinophil and polymorphonuclear leukocyte count was collected. All patients were premedicated with midazolam 0.02 mg/kg IV and received metoclopramide 10 mg and ranitidine 50 mg IV before induction. Anesthesia was induced with the same regimen in both groups: fentanyl 2 mcg/kg, propofol 2.5 mg/kg and rocuronium 0.8 mg/kg. When the BIS value was below 50 and the train of four (TOF) ratio was 0, direct laryngoscopy was performed, and the airway was secured with a flexible cuffed endotracheal tube of the appropriate size. Subsequently, capnography was applied, and the sevoflurane group was also monitored for minimum alveolar concentration (MAC). All patients were ventilated with protective lung ventilation with FiO_2_: 0.4 on air mixture, a tidal volume of 6–8 mL/kg of the ideal body weight and a positive end-expiratory pressure (PEEP) of 5 cm H_2_O. Partial pressure of end tidal CO_2_ (ETCO_2_) was maintained between 35–45 mmHg, while peak inspiratory pressure (Ppeak), plateau pressure (Ppl) and compliance were monitored.

Anesthesia maintenance was achieved either with a continuous infusion of propofol 1% (propofol group) or with sevoflurane (sevoflurane group) aiming at maintaining BIS values between 40 and 50. Muscle relaxation was maintained with bolus doses of rocuronium 0.3 mg/kg in order to maintain a TOF ratio < 3. The analgesic regimen consisted of fentanyl 3–5 mcg/kg/h, paracetamol 1 g, parecoxib 40 mg and tramadol 1 mg/kg intravenously. Intravenous ondansetron 4 mg was administered for postoperative nausea and vomiting prophylaxis approximately 30 min before recovery. Maintenance fluids were infused at a rate of 1 mL/kg/h.

During the operation, all parameters, such as heart rate (HR), systolic blood pressure (SBP), diastolic blood pressure (DBP), mean blood pressure (MAP), O_2_ saturation (SpO_2_), ETCO_2_, Ppeak, Ppl, PEEP, compliance, MAC and BIS, were recorded every 10 min.

On surgery completion, propofol infusion or sevoflurane inhalation were discontinued, residual muscle blockade was reversed with sugammadex 2 mg/kg and patients were extubated when spontaneous breathing was resumed, the TOF ratio was >90%, and there was response to verbal commands.

Patients were transferred to the Post Anesthesia Care Unit (PACU), where the second blood sample for eosinophil and polymorphonuclear leukocyte count was collected. When patients scored >8 on the modified Aldrete score, the second measurement of exhaled NO was performed (T_1_). As soon as patients scored >9 on the modified Aldrete score, with no postoperative nausea and vomiting, and a score < 3 on the pain Numeric Rating Scale (graded from 0: no pain to 10: worst pain imaginable), they were discharged from the PACU.

While on the ward, patients received paracetamol 1 g every 8 h, one dose of parecoxib (40 mg) and 1 mg/kg tramadol as rescue analgesia for acute pain.

At 24 h after the patients’ extubation, a third measurement of exhaled NO was performed (T_2_).

### 2.3. Study Endpoints

The primary endpoint of the study was to assess the alteration, if any, from the preoperative FeNO measurement (T_0_) to the immediate postoperative FeNO measurement (T_1_) in each group. Secondary outcomes of the study were the measurement of FeNO at 24 h after extubation (T_2_) in each group, the variation of eosinophil and polymorphonuclear leukocyte blood count immediately postoperatively in each group and the variation of Ppeak, Ppl and compliance in each group.

### 2.4. Statistical Analysis

Prior to this study, we conducted a pilot study on 10 patients per group in order to estimate the needed sample size for the detection of a significant fluctuation in the measurements of exhaled NO from the preoperative to the postoperative status. We demonstrated a mean drop of 5.5 ppb in the exhaled NO in the 10 patients of the propofol group and a mean drop of 5.3 ppb in the 10 patients of the sevoflurane group from the preoperative to the postoperative period. Therefore, by considering an average change of 5.4 ppb from the preoperative to the postoperative period to be clinically meaningful, and by hypothesizing a standard deviation (SD) of change of 10 ppb, we calculated that 29 patients should be included in each group in order to detect such a change with a power of 0.80 and an alpha error of 0.05. We enrolled 30 patients in each group to allow for drop-outs. The Kolmogorov–Smirnoff test was used to test the normality of distributions of the investigated parameters. Comparisons of numeric data between the two groups were performed with the unpaired *t*-test or the Mann–Whitney U-test for independent samples, depending on whether the variables followed a normal or non-normal distribution. The chi-square test or Fisher’s exact test, as appropriate, was used for comparisons of categorical data. Exhaled NO fluctuation in the two groups was analyzed by ANOVA for repeated measures and eosinophil and polymorphonuclear leukocyte variation within groups with the Wilcoxon signed rank test. Serial data (HR, SBP, DBP, MAP, SpO_2_, ETCO_2_, Ppeak, Ppl and compliance) were analyzed and compared between the two groups with a two-step summary measures technique [26]. Since there was variation in surgical times among patients, it was not deemed appropriate to compare mean values at every particular timepoint. In contrast, the area under the curve for values plotted against time was calculated for each patient, which was then divided by the number of recording points to provide one standardized value. Standardized data were then compared by intergroup analysis using unpaired *t*-test or Mann–Whitney U-test, as appropriate.

Data were expressed in terms of mean ± standard deviation (SD) or as median (25th–75th percentiles), depending on the normality of distributions for numeric variables and as number for categorical variables. For all statistical procedures, a value of *p* < 0.05 was considered statistically significant. Data were analyzed with IBM SPSS^®^ V21.0 for Windows (IBM Corp. Released 2012. IBM SPSS Statistics for Windows, Version 21.0. Armonk, NY, USA: IBM Corp.).

## 3. Results

**Figure 1:**
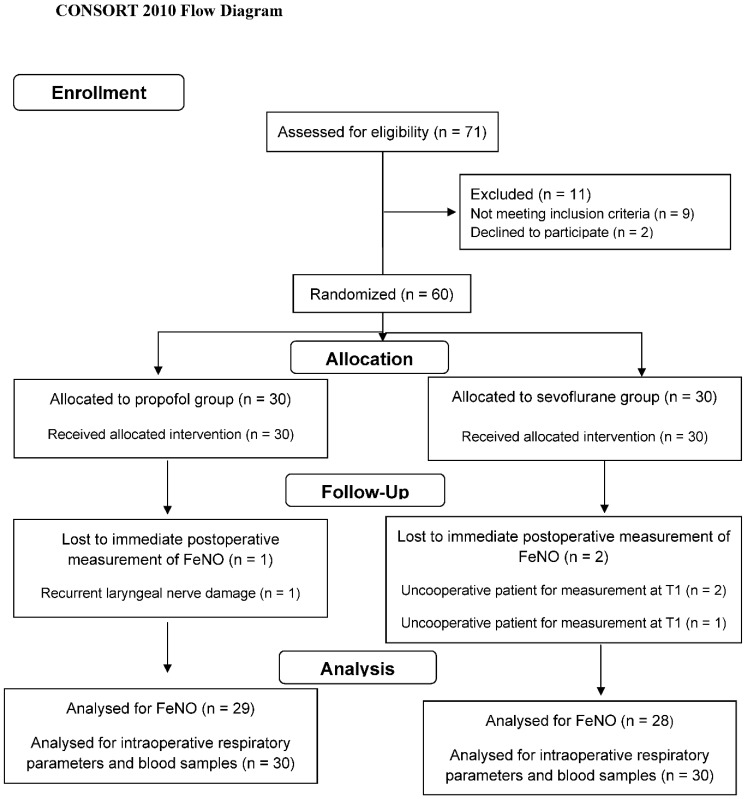
Study Flow Diagram.

Seventy-one patients were scheduled for total thyroidectomy with the same surgeon between May 2014 and April 2018. Sixty-two of them met the inclusion criteria, and two of them declined participation in the study. Therefore, 60 patients were enrolled. One patient in the propofol group was complicated by recurrent laryngeal nerve damage and was unable to perform the FeNO measurement at T_1_ and T_2_. Two patients in the sevoflurane group were unable to cooperate in order to perform the FeNO measurement at T_1_ (both of them) and at T_2_ (one of them). Central, lateral or bilateral lymph node dissection was not indicated in any of the patients with malignant conditions. The flowchart of the study including patient enrollment, allocation and analysis is presented in Figure 1. Patients’ demographics, surgery and anesthesia duration as well as baseline hemodynamic parameters were similar in the two groups (Table 1).

**Figure 2:**
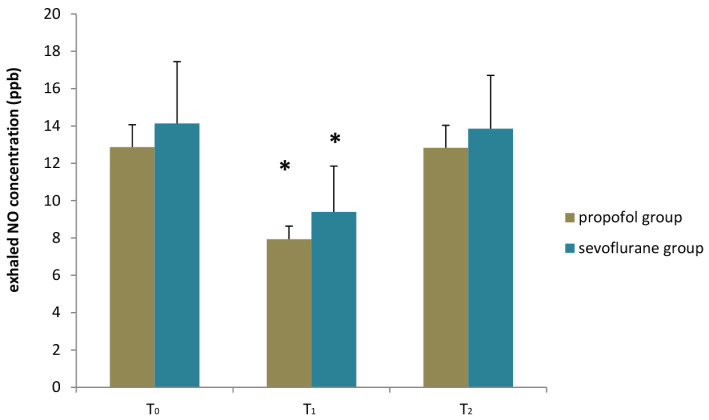
Exhaled NO fluctuation in the propofol and sevoflurane maintenance groups; * *p* < 0.05 in comparison to baseline (T_0_).

Both propofol and sevoflurane groups displayed decreased NO exhalation postoperatively (T_1_) in comparison to baseline values (T_0_) (7.93 ± 0.70 vs. 12.86 ± 1.19 ppb, *p* < 0.001 for the propofol group and 9.39 ± 2.45 vs. 14.13 ± 3.31 ppb, *p* < 0.001 for the sevoflurane group). Twenty-four hours postoperatively (T_2_), exhaled NO was no longer different from baseline in both the propofol and sevoflurane groups (12.83 ± 1.20 vs. 12.86 ± 1.19 ppb, *p* = 0.964 for the propofol group and 13.85 ± 2.85 vs. 14.13 ± 3.31 ppb, *p* = 0.535 for the sevoflurane group) (Figure 2).

**Figure 3:**
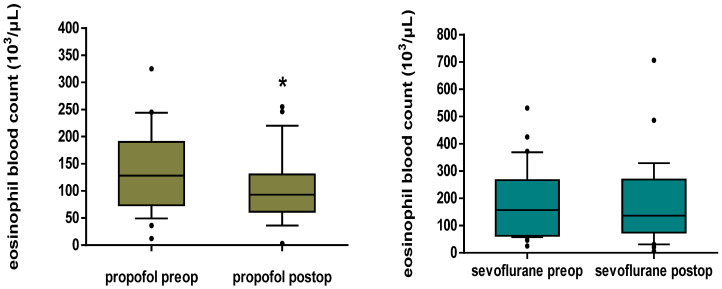
Box plots of eosinophil blood count in peripheral blood in the propofol and sevoflurane group preoperatively and postoperatively. Propofol maintenance caused a significant decrease in the eosinophil blood count (*p* < 0.001), while sevoflurane maintenance did not have a statistically significant effect (*p* = 0.746). The box plots depict the median and the interquartile range and the whiskers depict the 10th and 90th percentiles; * *p* < 0.05 in comparison to the preoperative status.

In the peripheral blood, a significant decrease in the eosinophil count was demonstrated in the propofol group postoperatively in comparison to baseline values (93.0 [63.7–129.7] vs. 128.0 [76.7–179.7] 10^3^/μL, *p* < 0.001). There was no significant difference in the eosinophil blood count postoperatively in comparison to baseline values in the sevoflurane group (136.0 [75.0–267.0] vs. 157.0 [62.0–267.0] 10^3^/μL, *p* = 0.746) (Figure 3).

**Figure 4:**
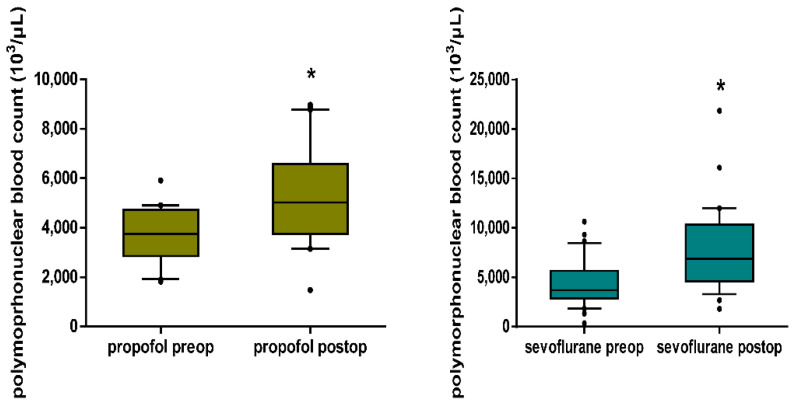
Box plots of polymorphonuclear blood count in peripheral blood in the propofol and sevoflurane group, preoperatively and postoperatively. Both propofol and sevoflurane maintenance caused a significant increase in the polymorphonuclear blood count (*p* < 0.001, respectively). The box plots depict the median and the interquartile range, and the whiskers depict the 10th and 90th percentiles; * *p* < 0.05 in comparison to the preoperative status.

Additionally, there was a significant increase in the polymorphonuclear blood count postoperatively in comparison to baseline in both the propofol and sevoflurane groups (5023.0 [3740.2–6465.2] vs. 3743.0 [2940.2–4707.2] 10^3^/μL, *p* < 0.001 for the propofol group and 6863.0 [4665.5–10314.5] vs. 3705.0 [2842.0–5656.0] 10^3^/μL, *p* < 0.001 for the sevoflurane group) (Figure 4).

The propofol group presented with lower standardized Ppeak and Ppl over time versus the sevoflurane group (16.6 ± 4.3 vs. 18.8 ± 3.4 cm H_2_O, *p* = 0.032 and 14.6 ± 4.2 vs. 16.6 ± 3.4 cm H_2_O, *p* = 0.054, respectively) and higher standardized compliance over time (42.4 [35.0–52.9] vs. 33.0 [30.0–41.5] mL/cm H_2_O, *p* = 0.027) (Table 2). Additionally, the propofol group had lower standardized heart rate over time versus the sevoflurane group (71.6 ± 7.7 vs. 76.6 ± 8.3 bpm, *p* = 0.019) and higher standardized DBP and MAP over time versus the sevoflurane group (76.8 ± 8.9 vs. 68.4 ± 8.8 mmHg, *p* < 0.001 and 93.2 ± 10.1 vs. 86.2 ± 9.2 mmHg, *p* = 0.007, respectively). Finally, the propofol group had lower standardized BIS values over time versus the sevoflurane group (45.2 ± 3.8 vs. 47.2 ± 2.6, *p* = 0.019). No differences between the two groups for standardized values of SBP, ETCO_2_ and SpO_2_ were demonstrated (Table 2).

None of the patients suffered allergic or anaphylactic reaction or bronchospasm, nor was any patient treated with exogenous nitric donors, such as nitroglycerin.

## 4. Discussion

According to the main results of this randomized controlled trial, anesthesia maintenance with either propofol or sevoflurane caused a significant reduction in immediate postoperative FeNO levels in adult patients subjected to thyroidectomy. This decrease was accompanied by a decrease in the postoperative eosinophil blood count as compared to the preoperative status, which, however, was significant only in the propofol group. Finally, respiratory lung mechanics seem to be better maintained in the propofol group as compared to the sevoflurane group, with the preservation of higher lung compliance and lower Ppeak and Pplateau over time in the former group.

We are not aware of other studies in the literature comparing the two modes of anesthetic maintenance as to NO exhalation in humans, which prompted us to undertake the current protocol. We selected only thyroidectomy patients in our study, where the operation was performed by the same experienced surgeon, mainly so that there would be consistency in both the surgeon and type of surgery and, as a result, manipulations and surgical stress would be similar for all patients, and secondly, because abdominal walls are not manipulated at all during thyroidectomy, and therefore, the postoperative measurement of exhaled NO would be literally painless for patients.

Since the early 1990s, when NO was first detected in exhaled breathing of all humans, its actions in the airway and the lungs have been constantly studied, as they reflect its versatile role as a bronchodilator, vasodilator, neurotransmitter and inflammatory response mediator [27]. In the early years, chemiluminescent analyzers were used in order to detect exhaled NO [27]. It was already at that time that FeNO was noted to be higher in asthmatic patients and to decrease in response to treatment with corticosteroids [28,29]. Currently, chemiluminescent analyzers are used mostly for laboratory analyses due to their greater size and weight, and electrochemical sensors have replaced them in clinical practice for the detection and reliable measurement of exhaled NO [30]. NObreath (Bedfont^®^ Scientific Ltd., Maidstone, UK), which we used in our study, is a portable, low-weight (≈400 g) analyzer with an electrochemical sensor, with FeNO measurement ranges from 5 to 500 ppb [31].

Even if FeNO does not seem to have the strength to confirm or rule out a diagnosis of asthma, as it can be elevated in non-asthma conditions or not elevated in some asthma phenotypes, it remains a useful tool for monitoring airway hyperresponsiveness and inflammation [24,32,33]. Particularly, when the FeNO level is co-evaluated with blood eosinophil count measurement, it has important specificity and sensitivity in predicting airway eosinophilia in asthma [34]. In addition, the measurement of FeNO, in combination with other risk assessment tools during the preanesthetic evaluation of patients with respiratory disease, might make the risk of perioperative and postoperative complications more predictable [35].

The significant reduction in NO exhalation in the propofol group in our study is in accordance with other studies reported in the literature, with, however, the vast majority of them dealing with NO measurement in the systemic circulation. Propofol, a safe and effective intravenous anesthetic routinely used for the induction and maintenance of anesthesia, also has a number of non-anesthetic effects related to NO activity. It displays an antioxidant potential by being able to directly scavenge hydroxyl chloride, superoxide, hydrogen peroxide and hydroxyl radicals and protect a variety of tissues from oxidant-related injury [36]. The antioxidant properties of propofol could be due to the fact that its chemical structure contains a phenolic hydroxyl group, which chemically resembles the antioxidant α-tocopherol [37]. Propofol can also protect macrophages from NO-induced cell death [38]. It has been shown that propofol enhances the activity of constitutive NO synthase (cNOS), but on the other hand, it inhibits the inducible production of NO both in vitro in experiments using whole blood from healthy volunteers and in surgical patients [39]. Propofol has also been found to have a direct inhibitory effect on iNOS expression in lipopolysaccharide-activated macrophages, thus downregulating the levels of NO production in macrophages [15,16]. Consequently, propofol may modify the excess production of NO and decrease the production of free radicals. These antioxidant and immunomodulating effects of propofol could contribute to the reduction in oxidative-related stress and inflammation in surgical patients [39].

Therefore, the reduction in FeNO levels in the propofol group in our study could be attributed to the direct action of propofol on the synthesis of iNOS isoform or to the inhibition of the release of inflammatory mediators from lung parenchyma, in accordance with the aforementioned findings. In fact, a reduction in the levels of such mediators by propofol has been demonstrated in in vivo experimental models, which have also demonstrated the protective effects of propofol on endotoxin-induced acute lung injury [17,18]. In fact, in a study by Chu et al., the attenuation by propofol of an endotoxin-induced increase in pro-inflammatory cytokines abrogated the microvascular leakage of protein and water in the lungs, thus preserving endothelial integrity [17]. In the same study, propofol significantly decreased exhaled NO and protein concentration in the bronchoalveolar lavage fluid, with its effects more evident in high doses. It was postulated by the authors that the protective action of propofol on endotoxin-induced acute lung injury could be mediated by the reduction in NO production. In a study by Gao et al., the early administration of propofol in rats subjected to endotoxin-induced acute lung injury resulted in reduced concentrations of nitrite/nitrate in the bronchoalveolar lavage fluid and attenuated iNOS expression in lung tissue [18]. In another study, propofol was able to reverse the oleic-acid-induced endothelial damage and subsequent inflammation and injury of lung parenchyma in conscious rats [40]. The authors suggested that NO production was involved in the oleic-acid-induced acute lung injury, since, in their study, an increase in exhaled NO and iNOS upregulation was noted in rats not treated with propofol, and these changes were reversed in propofol-treated animals. It could therefore be postulated that a similar pathophysiological mechanism of the suppression of the iNOS-NO-dependent pathway in the human lung parenchyma may underlie the decrease in FeNO levels by propofol, thus reducing the potential for inflammatory perturbation of the airway.

In our study, we also demonstrated a significant reduction in FeNO in the sevoflurane group. This is compatible with previous experimental studies investigating the effect of sevoflurane in several models of acute lung injury. In an experimental rat model of sepsis, pretreatment with sevoflurane attenuated sepsis-induced inflammatory response through a reduction in chemotactic cytokine levels and mitigated lipid peroxidation and oxidative stress [20]. It has already been shown that the induction of the expression of iNOS and, subsequently, overproduction of NO is implicated in the pathogenesis of acute lung injury in animals with endotoxemia [13,41,42]. In the Bedirli study, plasma NO levels were significantly reduced in comparison to the control group, prompting the authors to attribute decreased expression of inflammatory mediators in the sevoflurane group to the inhibition of intracellular NO-related signal transmission pathway [20]. Additionally, in an isolated buffer-perfused rat lung model, pretreatment with sevoflurane protected the lung against ischemia-reperfusion-induced injury, decreasing vascular permeability and reducing the production of NO metabolites in the perfusate [21]. This reduction indicates that the protective effects of sevoflurane against ischemia-reperfusion lung injury may be mediated through the inhibition of NO release. Both sevoflurane and isoflurane might share common pathways involving NO in the alleviation of acute lung injury, since, in an endotoxin-induced acute lung injury in rats, the proadministration of isoflurane resulted in the decreased pulmonary accumulation of proinflammatory cytokines, pulmonary nitrite/nitrate levels and significantly reduced iNOS gene expression in lung tissue [43]. It seems, therefore, that lung anti-inflammatory protection afforded by volatile anesthetics could be partially mediated through the inhibition of iNOS/NO pathway activation. Consequently, according to our results, sevoflurane seems to suppress the iNOS-dependent NO production in the human lung in a way similar to propofol, reducing the potential for inflammation.

In our study, polymorphonuclear blood count was increased in both groups. We consider that this response might be due to the effect of the surgical procedure. It is already known that immune responses after anesthesia and surgery are characterized by neutrophilia and that the surgical procedure plays a more important role than anesthesia per se in this response [44]. However, we demonstrated a significant decrease in eosinophil blood count postoperatively only in the propofol group. Eosinophils, after their release into the circulation, translocate into submucosal tissues, thus forming part of the immunological response at body surfaces, producing cytokines that influence acute and chronic inflammatory responses. The significant decrease in eosinophils in the propofol group is compatible with previous studies. Specifically, in a study examining the effect of various anesthetic agents on the chemotaxis of eosinophils in vitro, although the inhibition of eosinophilic chemotaxis was demonstrated only for thiopental and etomidate, the authors concluded that a similar effect for propofol could not be ruled out but could not be demonstrated due to the small power of the study [19]. Actually, in another study investigating a mouse model of allergic asthma, propofol significantly decreased the eosinophil count and the levels of proinflammatory mediators in the bronchoalveolar lavage fluid, attenuating infiltrating inflammatory cells and mucus production in histological samples [45]. The different response of eosinophils between propofol and sevoflurane anesthesia could be due to the different effect propofol and sevoflurane have on interleukin 10 (IL-10), whose role in the inflammatory response of the airway is a great significance. Il-10 has been found to be a strong inhibitor of eosinophil recruitment in mucosal tissue, contributing to the protection or resolution of airway inflammation in conditions such as asthma and chronic obstructive pulmonary disease [46,47,48]. In fact, the administration of propofol has been associated with reduced proinflammatory IL-6 levels and enhanced anti-inflammatory IL-10 generation as compared to sevoflurane, suggesting a more favorable anti-inflammatory effect of intravenous anesthesia in comparison to an inhalational technique [49]. This is obvious even in operations that require one-lung ventilation, which is a well-known factor to exert great stress on pulmonary function and homeostasis via the upregulation of proinflammatory cytokine expression either in systemic circulation or in the epithelial lining fluid [50,51]. In fact, there are several studies demonstrating that propofol anesthesia can more effectively suppress the perioperative inflammatory response as compared to inhalational techniques in operations involving one-lung ventilation [50,51,52].

An additional finding of our study was the more favorable respiratory profile and better preservation of lung mechanics in the propofol group, with demonstration of better compliance maintenance and favorable Ppeak and Ppl over time in comparison to the sevoflurane group. Sevoflurane has long been perceived as the preferable inhalational agent for anesthesia maintenance in patients suffering from asthma due to its favorable bronchodilatory effect [6]. However, when its use was studied in asthmatic children, the results were controversial [53], while on the other hand, propofol is also considered safe for asthmatic patients and has been shown to decrease airway resistance in patients with already hyperreactive airways [54,55]. It appears, therefore, that the expected increase in lung compliance due to bronchodilation caused by volatile anesthetics has perhaps been overestimated. In fact, animal experiments have shown that inhaled anesthetics inhibit the generation of lung surfactant by type II endothelial cells or reduce the efficacy of surfactant activity, thereby decreasing lung compliance [56]. Our finding of better respiratory mechanics over time with propofol maintenance are in accordance with an experimental study where sevoflurane maintenance resulted in significantly higher airway pressures than propofol during laparoscopy in a porcine model [57]. The protective effect of propofol against bronchoconstriction and increased respiratory resistance is perhaps mediated via the initiation of an anticholinergic mechanism during mechanical ventilation and resulting direct airway smooth muscle relaxant action, a fact that has been demonstrated in in vitro, experimental and human studies [58,59,60]. In fact, propofol anesthesia has been shown to decrease airway resistance even when no previous bronchoconstriction was present and was also associated with central airway dilatation observed at lung histology in a rat study [59]. The mechanism of the bronchodilatory effect of propofol remains to be elucidated, being perhaps associated with the inhibition of voltage-dependent calcium channels [61]. Propofol has also been shown to provide a dose-dependent relaxing effect in the chest wall muscles, affording an additional favorable effect on chest wall resistance [62,63,64]. A more potent muscle-relaxing effect of propofol versus sevoflurane at the same depth of anesthesia and a stronger relaxant action on airway muscles might account for its favorable effect on peak inspiratory pressures in our study.

Our study has a few limitations. First, we measured eosinophil blood count and not sputum eosinophil in our set of patients. Sputum eosinophil count has been long regarded as the most reliable indicator of eosinophilic airway inflammation. However, evolving evidence shows that eosinophil blood count seems to be equally reliable in predicting both eosinophilic airway inflammation and sputum eosinophil count [65]. It is important to note that the measurement of blood eosinophil count provides an easy sampling technique compared to the induction of sputum, particularly in the immediate postoperative period, because the latter would not only pose a risk of postoperative hemorrhage due to induced coughing but also create discomfort to the patient. Secondly, we can only make speculations about the aforementioned differential release of proinflammatory and anti-inflammatory cytokines between the two modes of anesthesia maintenance and the impact that these might have had on airway inflammation, because these were not measured in the current study. A further limitation is the fact that we did not evaluate the postoperative respiratory function of our patients via spirometry to confirm whether the aforementioned effects of the two anesthetic regimes on respiratory mechanics were sustained into the postoperative period. In addition, we did not correlate the preoperative volume of the thyroid gland with postoperative measurements of FeNO. Finally, we only enrolled patients without respiratory disease or airway hyperresponsiveness in our study, so it remains to be determined if our findings can be applied in those populations.

## 5. Conclusions

In conclusion, under the current study design, both propofol and sevoflurane maintenance techniques during thyroidectomy seem to decrease postoperative FeNO levels, with propofol additionally exerting a significant decrease in postoperative eosinophil blood count and providing a more favorable respiratory profile for the whole duration of the operation as compared to sevoflurane. The ease of measurement of FeNO by a portable device such as NObreath both for the anesthesiologist and the patient even in busy settings combined with its low cost could make it a useful tool in perianesthetic patient evaluation. According to our results, it appears that intravenous techniques may offer advantages in terms of the suppression of perioperative inflammatory perturbation in the local milieu of the airway as compared to inhalational techniques. Whether these findings can be extrapolated to patients with respiratory comorbidities or patients suffering from asthma or other forms of airway inflammation and hyperresponsiveness remains to be elucidated in future studies encompassing such patient populations.

## Figures and Tables

**Table 1:** Demographic and baseline hemodynamic characteristics of the two groups.

Variables	Propofol Group (*n* = 30)	Sevoflurane Group (*n* = 30)	*p* Value, Group Comparison
**Sex (M/F)**	5/25	4/26	1.000
**ASA (I/II/III)**	28/2/0	25/5/0	0.424
**Age (years), mean ± SD**	48.8 ± 12.0	49.3 ± 12.2	0.88
**Weight (kg), mean ± SD**	70.2 ± 13.9	76.1 ± 13.1	0.097
**Height (cm), mean ± SD**	164.7 ± 5.4	164.8 ± 5.9	0.910
**Smoking status (non-smoker/ex-smoker/smoker)**	30/0/0	26/4/0	0.117
**Indication for surgery;** Thyroid nodule/Hyperthyroidism/Substernal goiter/Thyroid cancer	8/6/11/5	12/1/9/8	0.153
**Duration of surgery (min), median [IQR]**	95.0 [75.0–105.0]	90.0 [75.0–105.0]	0.542
**Duration of anesthesia, (min), median [IQR]**	110.0 [90.0–130.0]	110.0 [100.0–120.0]	0.591
**Baseline SBP (mmHg), mean ± SD**	135.2 ± 21.1	137.1 ± 19.5	0.714
**Baseline DBP (mmHg), mean ± SD**	77.6 ± 8.1	74.2 ± 8.1	0.111
**Baseline MAP (mmHg), mean ± SD**	96.2 ± 11.3	93.7 ± 12.1	0.413
**Baseline HR (bpm), mean ± SD**	76.0 ± 8.7	78.6 ± 9.7	0.268

Abbreviations: SD, standard deviation; IQR, interquartile range; n, number; SBP, systolic arterial pressure; DBP, diastolic arterial pressure; MAP, mean arterial pressure; HR, heart rate.

**Table 2:** Standardized values for serial variables and comparisons between the two groups. * significant difference between groups.

Variables	Propofol Group (*n* = 30)	Sevoflurane Group (*n* = 30)	*p* Value, Group Comparison
**Standardized SBP over time (mmHg), mean ± SD**	123.4 ± 14.3	119.6 ± 11.2	0.260
**Standardized DBP over time (mmHg), mean ± SD**	76.8 ± 8.9	68.4 ± 8.8 *	**0.001**
**Standardized MAP over time (mmHg), mean ± SD**	93.2 ± 10.1	86.2 ± 9.2 *	**0.007**
**Standardized HR over time (bpm), mean ± SD**	71.6 ± 7.7	76.6 ± 8.3 *	**0.019**
**Standardized BIS over time, mean ± SD**	45.2 ± 3.8	47.2 ± 2.6 *	**0.019**
**Standardized SaO_2_ over time (%), mean ± SD**	98.7 ± 0.5	98.6 ± 0.6	0.301
**Standardized ETCO_2_ over time (mmHg), mean ± SD**	36.4 ± 2.9	36.5 ± 3.8	0.925
**Standardized Ppeak over time (cm H_2_O), mean ± SD**	16.6 ± 4.3	18.8 ± 3.4 *	**0.032**
**Standardized Ppl over time (cm H_2_O), mean ± SD**	14.6 ± 4.2	16.6 ± 3.4	0.054
**Standardized compliance over time (mL/cm H_2_O), median [IQR]**	42.4 [35.0–52.9]	33.0 [30.0–41.5] *	**0.027**

abbreviations: SD, standard deviation; IQR, interquartile range; SBP, systolic arterial pressure; DBP, diastolic arterial pressure; MAP, mean arterial pressure; HR, heart rate; Ppl, plateau pressure; bold is for significant differences.

## Data Availability

The datasets generated during and/or analyzed during the current study are available from the corresponding author on reasonable request.
